# HIV and the histopathologist

**DOI:** 10.4102/sajhivmed.v18i1.680

**Published:** 2017-04-24

**Authors:** Carolina E. Nel

**Affiliations:** 1 Department of Anatomical Pathology, National Health Laboratory Service, Wits University, South Africa

## Abstract

The practicing histopathologist is often a forgotten link in the management of HIV patients. This article aims to highlight the unique challenges faced by anatomical pathologists as well as focusing on the valuable contribution they can make to ensure prompt and accurate diagnoses that will ultimately benefit the patient.

## Introduction

As a practicing histopathologist working in the public sector, we are constantly exposed to cases where the patient is HIV-positive.

Often, pathologists are not seen as a crucial component in the treatment of the HIV-positive population.

This opinion piece serves to highlight the crucial and important role of a histopathologist in the management of these patients as well as look at the challenges we face in our daily practice.

## Clinical data

The presence of an adequate clinical history cannot be stressed enough. The patient’s retroviral disease (RVD) status, antiretroviral (ARV) status, CD4 count, viral load, drug history and presenting symptoms are crucial to provide a satisfying and useful pathology report. Without these essential data, the assessment of the patient’s tissue cannot be optimal.

Histopathologists need to know the clinical status of the patient to render an accurate and reproducible report. An important aspect of this clinical history on the patient’s request form includes the provision of a clinical differential diagnosis. This information will guide the pathologist as to whether the clinical assessment of the pathology correlates with the pathological findings. An example of this is a clinical history of a possible pyogenic granuloma that is submitted for microscopic evaluation. The histological diagnosis of Kaposi’s sarcoma by the pathologist would not be inconsistent with this clinical diagnosis, and the pathologist would be comfortable in authorising the report.

Another important aspect of a complete set of clinical data is the possibility of research that can be conducted in this sphere. Academic research is essential to ensure improved management of the HIV-positive population. A complete set of clinical data will aid in providing clinically significant results. Incomplete clinical information may hamper the impact that pathological findings may have in contributing to optimum treatment or management protocols. To have real clinical significance and impact, clinicians and pathologists should be embarking on prospective studies in our population as we are uniquely positioned in the public sector to provide guidance and academic data from which evidence-based medicine can be practiced. We should be providing the world with high-quality research material on HIV by collaborating with our clinical colleagues on a regular basis.

## Cut up

The discipline of anatomical pathology is a multi-step process with different individuals performing different tasks. The first contact with the specimen is in the laboratory during macroscopic assessment. The challenge for the anatomical pathologist, therefore, starts at the cut up of specimens where trainees, pathologists and laboratory staff are exposed to human tissue. The macroscopic evaluation of a specimen is an important adjunct to the assessment on the microscopic level. Using knives and sharp equipment exposes laboratory staff to the virus. Knowledge regarding the patient’s immune status becomes essential to ensure personal protective equipment is worn, and laboratory guidelines regarding potential infective agents are adhered to. Providing the pathologist in the laboratory with the HIV status on the patient’s request form will alert them to look for specific HIV-related findings, for example, the presence or absence of caseous necrosis that can be observed macroscopically in lymph nodes or other organs. This finding can result in the immediate request of a Ziehl–Neelsen stain, which will speed up the diagnosis of tuberculosis.

## Ethical issues

Should a pathologist raise the possibility of immunocompromise in his or her report if the patient’s HIV status is unknown? Is it ethical not to mention this possibility? This is not a common scenario but becomes problematic as it may affect clinical decision-making and future management of the patient. Personally, I feel that a histopathologist has a duty to the clinician and the patient to raise the issue of possible immunocompromise albeit not of HIV *per se*. This is where the histopathologist can assist in the multidisciplinary management of these patients. The role of a pathologist in the interdisciplinary meetings is of great value.

## Double pathology

In the era of HIV, the presence of double pathology has become a significant factor in our daily practice. The HIV-positive population are not just at risk of developing opportunistic infections but are also exposed to the usual risk factors of other malignancies and diseases of that specific gender and age group. Often, the clinician is unsuspecting of double pathology, for example, lymphoma with a superimposed Kaposi’s sarcoma or tuberculosis. This is not uncommon in our daily practice.

## Diagnostic challenges

The introduction of antiretroviral therapy (ART) has changed not just the lives of clinicians but also those of the practicing histopathologist. This has resulted in a change in the pathology of diseases and evolution of infectious diseases. We have seen a significant increase in cases of syphilis, for example, which is a disease known to have a diverse clinical presentation. The explanation for this apparent increase may be twofold. The first is a higher index of suspicion for the infection from a diagnostic point of view. The second is the immune reconstitution inflammatory syndrome (IRIS) phenomenon that may unmask latent diseases including syphilis, tuberculosis and cryptococcosis. The availability of the immunohistochemical stain in the laboratory for routine detection of *Treponema pallidum* organisms has contributed to the higher diagnostic yield of this disease. The histological presentation of syphilis remains non-specific, but a vigilant pathologist with a sharp clinician will result in an accurate diagnosis of this treatable infection.

The diagnosis of many disease processes has been complicated by the HIV epidemic, but none as severe as its impact on haematolymphoid pathology. Lymphomas driven by Ebstein-Barr virus (EBV) and human herpes virus-8 (HHV-8) are often difficult to classify adequately because of the deranged immune system in these patients. The grey zone between a reactive lymphoid population and a true haematolymphoid neoplasm has increased and remains a massive diagnostic challenge.

## Prognostic value

An important part of the management of the HIV-infected population is follow-up histology to assess the response to treatment or to assess disease progression. Pathologists are in a unique position to assist clinicians in this regard. For instance, the persistence of squamous intra-epithelial lesions in the cervix is an example where pathologists can assist in decision-making regarding patient’s need for large loop excision of the transformation zone (LLETZ) procedures or hysterectomies.

Pathological findings are important not only from a diagnostic perspective but also from a prognostic point of view. Histopathologists are able to provide essential information in a report regarding prognosis of a disease. Assessment for bone marrow involvement is a useful tool used for staging purposes in many malignancies including lymphomas, carcinomas and other neoplasms such as small round blue cell tumours in children. An excellent example of prognostic value add in histopathology is the use of Ki-67 (proliferation marker) in the assessment of lymphomas. A high proliferation index will indicate a more aggressive tumour with a possibly worse prognosis and resistance to standard chemotherapy. Histologic grading of malignancies is also a function of the histopathologist which in turn has a direct impact on staging, prognosis and management protocols.

## The future

Pathologists in South Africa are a crucial but often unrecognised part of the management team of the HIV population. We can add valuable research value in this area of HIV management and provide input with regard to disease progression, side effects of drugs and assist in diagnosis. The pathologist is often able to provide a quick and accurate provisional result in infective conditions long before culture results are available.

Antiretroviral therapy treatment has increased the life expectancy of HIV-positive patients. This has resulted in an emergence of a new spectrum of diseases not previously encountered in this population. A new era of paediatric HIV patients will also provide new challenges as certain disease processes have not been studied in this group.

## Conclusion

The histopathologist is perfectly positioned to assist in the management of both acute and chronic illnesses in the context of the HIV-infected patient. An accurate and detailed clinical history as well as a sound relationship with the treating clinician will go a long way in improving patient care.

